# Patient-reported outcomes from a randomized phase III trial of sarilumab monotherapy versus adalimumab monotherapy in patients with rheumatoid arthritis

**DOI:** 10.1186/s13075-018-1614-z

**Published:** 2018-06-19

**Authors:** Vibeke Strand, Laure Gossec, Clare W. J. Proudfoot, Chieh-I Chen, Matthew Reaney, Sophie Guillonneau, Toshio Kimura, Janet van Adelsberg, Yong Lin, Erin K. Mangan, Hubert van Hoogstraten, Gerd R. Burmester

**Affiliations:** 1 Stanford University, Palo Alto, CA USA; 2 Sorbonne Universités, UPMC Université Paris 6, GRC-UPMC 08 (EEMOIS), Paris, France; 3 Department of Rheumatology, Assistance publique – Hôpitaux de Paris, Pitié Salpêtrière Hospital, Paris, France; 4 Sanofi, Guildford, UK; 5 Regeneron Pharmaceuticals, Inc., 777 Old Saw Mill River Road, Tarrytown, NY 10591-6707 USA; 6 Sanofi, Paris, France; 7 Sanofi, Bridgewater, NJ USA; 8 Charité - University Medicine, Berlin, Germany; 9 Present Address: ViiV Healthcare, Brentford, UK; 10 Present Address: Celgene, Summit, NJ USA

**Keywords:** Sarilumab, Adalimumab, Biologic disease-modifying antirheumatic drugs, Rheumatoid arthritis, Patient-reported outcomes

## Abstract

**Background:**

The phase III MONARCH randomized controlled trial (NCT02332590) demonstrated that in patients with rheumatoid arthritis (RA), sarilumab (anti-interleukin-6 receptor monoclonal antibody) monotherapy is superior to adalimumab monotherapy in reducing disease activity and signs and symptoms of RA, as well as in improving physical function, with similar rates of adverse and serious adverse events. We report the effects of sarilumab versus adalimumab on patient-reported outcomes (PROs).

**Methods:**

Patients with active RA intolerant of, or inadequate responders to, methotrexate were randomized to sarilumab 200 mg plus placebo every 2 weeks (q2w; *n* = 184) or adalimumab 40 mg plus placebo q2w (*n* = 185). Dose escalation to weekly administration of adalimumab or matching placebo was permitted at week 16. PROs assessed at baseline and weeks 12 and 24 included patient global assessment of disease activity (PtGA), pain and morning stiffness visual analogue scales (VASs), Health Assessment Questionnaire Disability Index (HAQ-DI), 36-item Short Form Health Survey (SF-36), Functional Assessment of Chronic Illness Therapy–Fatigue (FACIT-F), Rheumatoid Arthritis Impact of Disease (RAID), and rheumatoid arthritis-specific Work Productivity Survey (WPS-RA). Between-group differences in least-squares mean (LSM) changes from baseline were analyzed. *p* < 0.05 was considered significant for PROs in a predefined hierarchy. For PROs not in the hierarchy, nominal *p* values are provided. Proportions of patients reporting improvements greater than or equal to the minimal clinically important difference (MCID) and achieving normative values were assessed.

**Results:**

At week 24, sarilumab treatment resulted in significantly greater LSM changes from baseline than adalimumab monotherapy in HAQ-DI (*p* < 0.005), PtGA (*p* < 0.001), pain VAS (*p* < 0.001), and SF-36 Physical Component Summary (PCS) (*p* < 0.001). Greater LSM changes were reported for sarilumab than for adalimumab in RAID (nominal *p* < 0.001), morning stiffness VAS (nominal *p* < 0.05), and WPS-RA (nominal *p* < 0.005). Between-group differences in FACIT-F and SF-36 Mental Component Summary (MCS) were not significant. More patients reported improvements greater than or equal to the MCID in HAQ-DI (nominal *p* < 0.01), RAID (nominal *p* < 0.01), SF-36 PCS (nominal *p* < 0.005), and morning stiffness (nominal *p* < 0.05), as well as greater than or equal to the normative values in HAQ-DI (*p* < 0.05), with sarilumab versus adalimumab.

**Conclusions:**

In parallel with the clinical efficacy profile previously reported, sarilumab monotherapy resulted in greater improvements across multiple PROs than adalimumab monotherapy.

**Trial registration:**

ClinicalTrials.gov, NCT02332590. Registered on 5 January 2015.

**Electronic supplementary material:**

The online version of this article (10.1186/s13075-018-1614-z) contains supplementary material, which is available to authorized users.

## Background

Interleukin-6 (IL-6) signaling plays a key role in mediating the underlying disease pathophysiology and clinical manifestations of rheumatoid arthritis (RA), including pain and fatigue [1–5]. Sarilumab is an investigational novel human immunoglobulin G1 (IgG1) monoclonal antibody that inhibits IL-6-mediated signaling by selectively binding to soluble and membrane-bound IL-6 receptor α [6].

Patients report a significant number of adverse events (AEs) associated with conventional synthetic disease-modifying antirheumatic drugs (csDMARDs), particularly with methotrexate (MTX) [7, 8]. Consequently, at least 30% of patients with RA using biologic DMARDs (bDMARDs) do not use concomitant background therapy, highlighting the importance of bDMARD monotherapy as a therapeutic option [9, 10]. As previously reported, sarilumab in combination with csDMARDs reduces disease activity and improves patient-reported outcomes (PROs) with a manageable safety and tolerability profile consistent with IL-6 receptor blockade [6, 11–13]. The superior efficacy of sarilumab monotherapy in comparison with adalimumab monotherapy was shown in the phase III, multicenter, randomized controlled trial (RCT) MONARCH (NCT02332590) [14]. Rates of AEs and serious AEs observed in MONARCH were similar between treatment groups. Although neutropenia was more common with sarilumab, infection rates were comparable between groups [14].

PROs supplement physician-reported clinical assessments and acute-phase reactant measurements, and they present a more complete understanding of disease, treatment, and treatment impact on patients. Using the American College of Rheumatology (ACR) core dataset alone to define disease activity and treatment benefit does not account for other disease symptoms that are important to patients with RA or for the significant physical, social, and psychological impacts of RA on a patient’s daily life and functioning [15–19]. Quantifying fatigue, the effect of RA symptoms on participation (work within and outside the home, family, and social and leisure activities), and ultimately on broad health status or health-related quality of life (HRQOL), is vital for a comprehensive evaluation of RA treatments such as sarilumab [20]. PROs, measured by validated and reliable instruments in appropriately designed trials, offer a means to obtain these data in a clinically meaningful way [21]. This paper reports our comparison of PROs of patients with active RA who received sarilumab monotherapy vs. adalimumab monotherapy.

## Methods

### Study design and patient population

Details of the MONARCH trial have been published previously [14]. Briefly, patients (aged ≥ 18 years) with active RA who were either intolerant of or inadequate responders (IR) to an MTX dose of 10–25 mg/wk, or 6–25 mg/wk for patients within the Asia-Pacific region, or considered inappropriate for MTX treatment by investigator judgment were eligible for inclusion. Active RA was defined as at least six swollen and at least eight tender joints, high-sensitivity C-reactive protein ≥ 8 mg/L or erythrocyte sedimentation rate (ESR) ≥ 28 mm/h, and 28-joint Disease Activity Score (DAS28)-ESR > 5.1, with disease duration ≥ 3 months. Patients were randomized to receive sarilumab 200 mg plus placebo every 2 weeks (q2w) or adalimumab 40 mg plus placebo q2w, administered subcutaneously for 24 weeks. After week 16, dose escalation to weekly administration of adalimumab or matching placebo was permitted for patients who did not achieve ≥ 20% improvement in tender and swollen joint counts. The protocol was approved by appropriate ethics committees/institutional review boards, and all patients provided written consent prior to study participation. The trial was conducted in compliance with institutional review board regulations, the International Conference on Harmonisation Guidelines for Good Clinical Practice, and the Declaration of Helsinki.

### Patient-reported outcomes

PROs included patient global assessment of disease activity (PtGA) [22], pain [22] (both evaluated using a 0- to 100-mm visual analogue scale [VAS]), and Health Assessment Questionnaire Disability Index (HAQ-DI) to measure physical functioning [22], all of which were included as part of the ACR core set. The 36-item Short Form Health Survey (SF-36) measures health status (or HRQOL) [23] and assesses eight domains on a scale of 0–100, with higher scores indicating better health status: physical functioning (PF), role physical (RP), bodily pain (BP), general health (GH) perceptions, vitality (VT), social functioning (SF), role emotional (RE), and mental health (MH). These domains are also combined into Physical Component Summary (PCS) and Mental Component Summary (MCS) scores. Other PROs included the Functional Assessment of Chronic Illness Therapy–Fatigue (FACIT-F) scale [24]; morning stiffness, measured by its severity on a 0-mm (no problem) to 100-mm (major problem) VAS [25]; the Rheumatoid Arthritis Impact of Disease (RAID) questionnaire [26]; and the RA-specific Work Productivity Survey (WPS-RA) [27]. The RAID is a composite measure based on seven domains (pain, functional disability, fatigue, physical and emotional well-being, quality of sleep, and coping) scored on 0–10 numerical rating scales, with lower scores indicative of less disease impact [26]. The WPS-RA consists of nine items assessing employment status, absenteeism (days missed), presenteeism (days with work productivity reduced by ≥ 50%), rate of RA interference in work within and outside the home (0 = no interference to 10 = complete interference), and participation measured as days missed in family, leisure, and social activities [27]. All PROs were assessed at baseline and weeks 12 and 24.

### Statistical analyses

Analyses were based on the intention-to-treat (ITT) population. For continuous endpoints, between-group differences in changes from baseline were determined using a mixed-model approach for repeated measures, with treatment, visit, treatment-by-visit interaction, and region as fixed effects and the corresponding baseline PRO scores as continuous covariates. Data collected after patients had their dose of adalimumab (or matching placebo) increased were included in the primary analysis; data collected after permanent treatment discontinuation of either sarilumab or adalimumab were excluded. Statistical significance could be claimed for those outcomes above the break in the predefined statistical analysis hierarchy, which included changes from baseline to week 24 in HAQ-DI and SF-36 PCS scores. Because PtGA and pain VAS were part of the ACR20/50/70 core set, a prespecified secondary efficacy endpoint, they were also considered above the hierarchy break. For endpoints below the hierarchy break or not included in the hierarchy, *p* values are considered nominal. Because the WPS-RA consists of independent items, O’Brien’s global test was first used to determine overall significance at week 24 prior to further evaluation.

The proportion of patients reporting clinically meaningful improvements, such as meeting or exceeding published values for minimal clinically important differences (MCIDs) (“responders”), was evaluated for each PRO. These were prespecified for HAQ-DI (≥ 0.22 units [28] and ≥ 0.3 units [29, 30]) and applied post hoc for PtGA, pain and morning stiffness VAS (≥ 10 units [31, 32]), SF-36 (≥ 2.5 units for PCS and MCS and ≥ 5 units for domains [33]), FACIT-F (≥ 4 units [24]), and RAID (≥ 3 units [34]). Patients who discontinued treatment were considered nonresponders in the analyses. Between-group differences in proportions were analyzed using the Cochran-Mantel-Haenszel test stratified by region. Additional post hoc analyses included the proportion of patients reporting scores greater than or equal to normative values for the U.S. general population in HAQ-DI (< 0.05) [35], FACIT-F (≥ 40.1) [36], SF-36 PCS and MCS (≥ 50), and age- and sex-matched SF-36 domain scores at week 24.

## Results

### Patient demographics and baseline characteristics

Detailed patient demographics and baseline disease characteristics have been described previously [14]. In brief, the ITT population consisted of 369 patients (sarilumab, *n* = 184; adalimumab, *n* = 185). Baseline characteristics as well as disease and treatment history were generally balanced between treatment groups (Table 1). The mean (SD) age of participants was 52.2 (12.3) years; most were female (83%) and white (91%). Mean (SD) durations of RA were 8.1 (8.1) years in the sarilumab group and 6.6 (7.8) years in the adalimumab group; mean (SD) baseline DAS28-ESR was 6.8 (0.8) in both groups. Baseline PRO scores were generally balanced between treatment groups (Table 2). At baseline, 78 (42.6%) patients in the sarilumab group and 69 (37.5%) in the adalimumab group were employed outside the home. Six patients in the adalimumab group and five patients in the sarilumab group who met protocol criteria received dose escalations of weekly administration of adalimumab (adalimumab group) or matching placebo (sarilumab group). The study was completed by most patients (sarilumab, 90%; adalimumab, 84%), with AEs being the most common cause of discontinuation.

**Table 1:** Patient demographics and disease characteristics

	Sarilumab SC 200 mg q2w (*n* = 184)	Adalimumab SC 40 mg q2w (*n* = 185)
Demographics		
Age, years, mean ± SD	50.9 ± 12.6	53.6 ± 11.9
Female sex, *n* (%)	157 (85.3)	150 (81.1)
Race, white, *n* (%)	171 (92.9)	164 (88.6)
Weight, kg, mean ± SD	72.3 ± 16.5	71.8 ± 17.8
BMI, kg/m^2^, mean ± SD	27.1 ± 5.6	27.3 ± 6.5
Geographic region, *n* (%)^a^		
1	61 (33.2)	62 (33.5)
2	36 (19.6)	35 (18.9)
3	87 (47.3)	88 (47.6)
Disease and treatment history		
Duration of RA, years, mean ± SD	8.1 ± 8.1	6.6 ± 7.8
Rheumatoid factor-positive, *n* (%)^b^	119 (66.9)	116 (64.8)
Anti-CCP autoantibody-positive, *n* (%)^c^	134 (75.3)	138 (76.7)
Number of prior csDMARDs, *n* (%)		
0	0	0
1	83 (45.1)	88 (47.6)
2	57 (31.0)	58 (31.4)
≥ 3	44 (23.9)	39 (21.1)
Prior csDMARDs other than MTX, *n* (%)^d^		
Sulfasalazine	59 (32.1)	44 (23.8)
Leflunomide	42 (22.8)	45 (24.3)
Hydroxychloroquine	41 (22.3)	43 (23.2)
Prior csDMARDs in combination with MTX, *n* (%)	35 (19.0)	44 (23.8)
Reason for stopping MTX, *n* (%)		
Inadequate responder	97 (52.7)	103 (55.7)
Intolerant	87 (47.3)	81 (43.8)
Inappropriate for continued treatment	0	1 (0.5)
Concomitant oral corticosteroids, *n* (%)	98 (53.3)	104 (56.2)
Disease activity, mean ± SD		
DAS28-ESR^e^	6.8 ± 0.8	6.8 ± 0.8
DAS28-CRP^e^	6.0 ± 0.9	6.0 ± 0.9
Swollen joint count (66 assessed)^e^	18.6 ± 10.7	17.5 ± 10.3
Tender joint count (68 assessed)^e^	28.0 ± 13.2	26.7 ± 13.6
CDAI^e^	43.6 ± 12.1	42.4 ± 12.0
ESR, mm/h^e^	46.5 ± 21.8	47.5 ± 23.2
CRP, mg/L^e^	17.4 ± 21.3	24.1 ± 31.0

*Abbreviations: BMI* Body mass index, *CCP* Cyclic citrullinated peptide, *CDAI* Clinical Disease Activity Index, *CRP*, C-reactive protein, *csDMARD* Conventional synthetic disease-modifying antirheumatic drug, *DAS28* 28-joint Disease Activity Score, *ESR* Erythrocyte sedimentation rate, *MTX* Methotrexate, *q2w* Every 2 weeks, *RA* Rheumatoid arthritis, *SC* Subcutaneous

^a^Region 1 (Western countries): Czech Republic, Germany, Hungary, Israel, Spain, United States. Region 2 (South America): Chile, Peru. Region 3 (rest of the world): Poland, South Africa, South Korea, Romania, Russia, Ukraine

^b^Adalimumab group, *n* = 179; sarilumab group, *n* = 178

^c^Adalimumab group, *n* = 180; sarilumab group, *n* = 178

^d^Included if used in > 5% of the population

^e^Higher numbers represent more severe disease

**Table 2:** Patient-reported outcome scores at baseline (intention-to-treat population)

Patient-reported outcome	Sarilumab SC 200 mg q2w (*n* = 184)	Adalimumab SC 40 mg q2w (*n* = 185)
HAQ-DI^a^	1.6 (0.55)	1.6 (0.64)
Pain VAS^b^	71.6 (18.65)	71.4 (18.96)
PtGA^b^	68.0 (17.49)	67.8 (18.41)
SF-36^c^		
PCS	30.7 (6.15)	31.4 (6.59)
MCS	36.7 (10.67)	37.1 (11.83)
Physical functioning	33.3 (20.04)	35.7 (21.73)
Role physical	34.4 (18.88)	34.2 (20.08)
Bodily pain	26.7 (14.75)	28.4 (16.55)
General health	34.4 (15.64)	36.1 (16.21)
Vitality	33.4 (16.42)	35.5 (17.87)
Social functioning	46.5 (23.23)	47.4 (26.03)
Role emotional	47.1 (24.44)	47.4 (27.25)
Mental health	49.0 (18.52)	50.5 (19.69)
FACIT-F^d^	23.6 (9.01)	24.0 (10.31)
Morning stiffness VAS^b^	70.8 (18.99)	68.0 (21.37)
RAID score (0–10)^e^	6.7 (1.72)	6.4 (2.03)
WPS-RA		
Employed outside the home, *n* (%)	78 (42.6)	69 (37.5)
Work days missed	2.5 (5.26)	2.0 (5.11)
Days with work productivity reduced by ≥ 50%	5.8 (7.03)	4.9 (7.66)
Interference with work productivity^f^	5.6 (2.62)	4.8 (2.98)
Housework days missed	8.7 (8.14)	7.3 (9.06)
Days with household productivity reduced by ≥ 50%	10.0 (8.78)	9.4 (9.68)
Interference with household productivity	6.5 (2.60)	6.3 (2.88)
Days with family, social, or leisure activities missed	5.4 (7.84)	5.6 (8.51)
Days with outside help hired	5.2 (8.43)	4.6 (8.57)

*Abbreviations: FACIT-F* Functional Assessment of Chronic Illness Therapy–Fatigue; *HAQ-DI* Health Assessment Questionnaire Disability Index, *MCS* Mental Component Summary, *PCS* Physical Component Summary, *PtGA* Patient global assessment of disease activity, *q2w* Every 2 weeks, *RAID* Rheumatoid Arthritis Impact of Disease, *SC* Subcutaneous, *SF-36* 36-item Short Form Health Survey, *VAS* Visual analogue scale, *WPS-RA* Rheumatoid arthritis-specific Work Productivity Survey

^a^Scale range, 0–3; lower scores represent less difficulty with physical functioning

^b^Scale range, 0–100; lower scores indicate better outcomes

^c^Scale range, 0–100; higher scores represent less impaired physical/mental health status

^d^Scale range, 0–52; higher scores represent less fatigue

^e^Scale range, 0–10; higher scores indicate a greater (negative) impact of RA

^f^Scale range, 0 = no interference to 10 = complete interference

### Changes from baseline at weeks 12 and 24

**Fig. 1:**
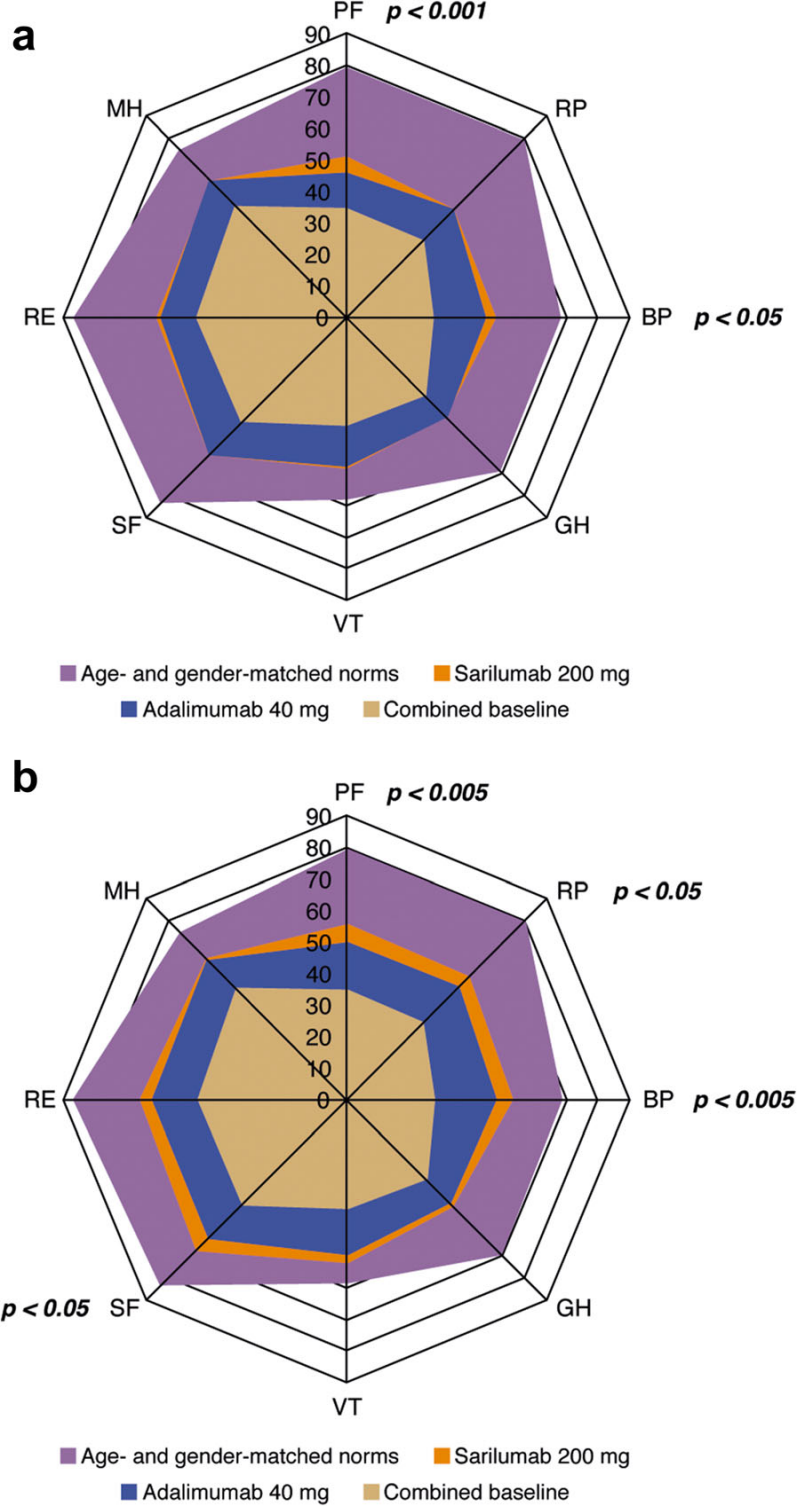
Baseline and posttreatment scores at week 12 (**a**) and week 24 (**b**) across all 36-item Short Form Health Survey domains compared with age- and sex-matched U.S. general population norms. *BP* Body pain, *GH* General health, *MH* Mental health, *PF* Physical functioning, *RE* Role emotional, *RP* Role physical, *SF* Social functioning, *VT* Vitality. *p* Values show the least-squares mean between-group difference in the change from baseline. *p* Values provided for week 12 are nominal. Because combined baseline scores are presented, change from baseline for each group cannot be inferred from the figure alone

Table 3 summarizes least-squares mean (LSM) changes in PROs reported with sarilumab 200 mg q2w and adalimumab 40 mg q2w from baseline to week 24. Top-line results for HAD-QI, PtGA, SF-36, FACIT-F, and pain VAS have been reported previously [14], but they are presented here in detail for completeness. At week 24, both sarilumab and adalimumab treatment resulted in improvements in all PROs. LSM changes from baseline in HAQ-DI (*p* < 0.005), pain VAS (*p* < 0.001), PtGA (*p* < 0.001), and SF-36 PCS scores (*p* < 0.001) were significantly greater with sarilumab than with adalimumab; however, the SF-36 MCS scores were not statistically different (*p* = 0.332). At week 24, improvements in four of eight SF-36 domains were significantly greater with sarilumab than with adalimumab (PF and BP, both *p* < 0.005; RP and SF, both *p* < 0.05) (Fig. 1 and Table 3). At week 24, LSM changes in FACIT-F were not statistically different (*p* = 0.069) (Table 3).

**Table 3:**
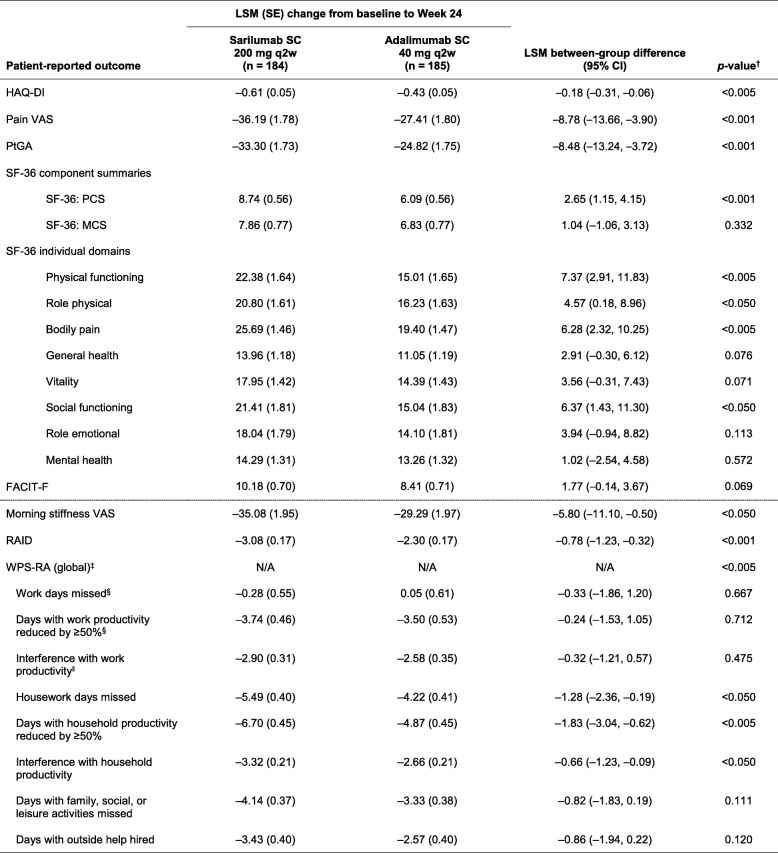
Change from baseline to week 24 with sarilumab 200 mg or adalimumab 40 mg every 2 weeks

*Abbreviations: ACR* American College of Rheumatology; *FACIT-F* Functional Assessment of Chronic Illness Therapy–Fatigue; *HAQ-DI* Health Assessment Questionnaire Disability Index; *LSM* Least-squares mean; *MCS* Mental Component Summary; *N/A* Not applicable; *PCS* Physical Component Summary; *PtGA* Patient global assessment of disease activity; *q2w* Every 2 weeks; *RAID* Rheumatoid Arthritis Impact of Disease; *SC* Subcutaneous; *SF-36* 36-item Short Form Health Survey; *VAS* Visual analogue scale; *WPS-RA* Rheumatoid arthritis-specific Work Productivity Survey

^a^LSM between-group differences (sarilumab vs. adalimumab). All *p* values below the solid line are nominal (*see* the Methods section of text)

^b^Global test for the change from baseline in the eight WPS-RA scores

^c^Number of patients included in the analysis for this element of the WPS-RA score: adalimumab group, *n* = 60; sarilumab group, *n* = 70

^d^Number of patients included in this element of the WPS-RA score: adalimumab group, *n* = 57; sarilumab group, *n* = 68

At week 24, improvements in morning stiffness VAS were greater with sarilumab treatment than with adalimumab (nominal *p* < 0.05), as were improvements in total RAID score (nominal *p* < 0.001) and all seven individual RAID components (all nominal *p* < 0.05), with greatest effects observed in the pain and functional disability domains (Additional file 1: Table S1). At week 24, global testing indicated that sarilumab treatment resulted in greater overall improvement in WPS-RA than adalimumab (nominal *p* < 0.005), including more reductions in the number of household work days missed due to RA (nominal *p* < 0.05), days in which household productivity was reduced by ≥ 50% (nominal *p* < 0.005), and the rate of RA interference with household work (nominal *p* < 0.05).

LSM changes reported at week 12 are summarized in Additional file 1: Table S2. Similar results were reported at week 12 for all PROs; however, LSM between-group differences increased over time, with greater differences reported at week 24 than at week 12 for all PROs.

As part of the study, patients who did not achieve a ≥ 20% improvement in tender and swollen joint counts were permitted to escalate their dose to weekly administration of adalimumab or matching placebo. A total of five patients in the sarilumab group and six patients in the adalimumab group met the requirements for dose escalation. A sensitivity analysis was conducted where all data after treatment discontinuation or adalimumab (or matching placebo) dose increase were set to missing and a multiple imputation approach was used. A statistically significant difference in favor of sarilumab compared with adalimumab was observed, which was similar with that in the primary analysis.

### Responder analysis

**Fig. 2:**
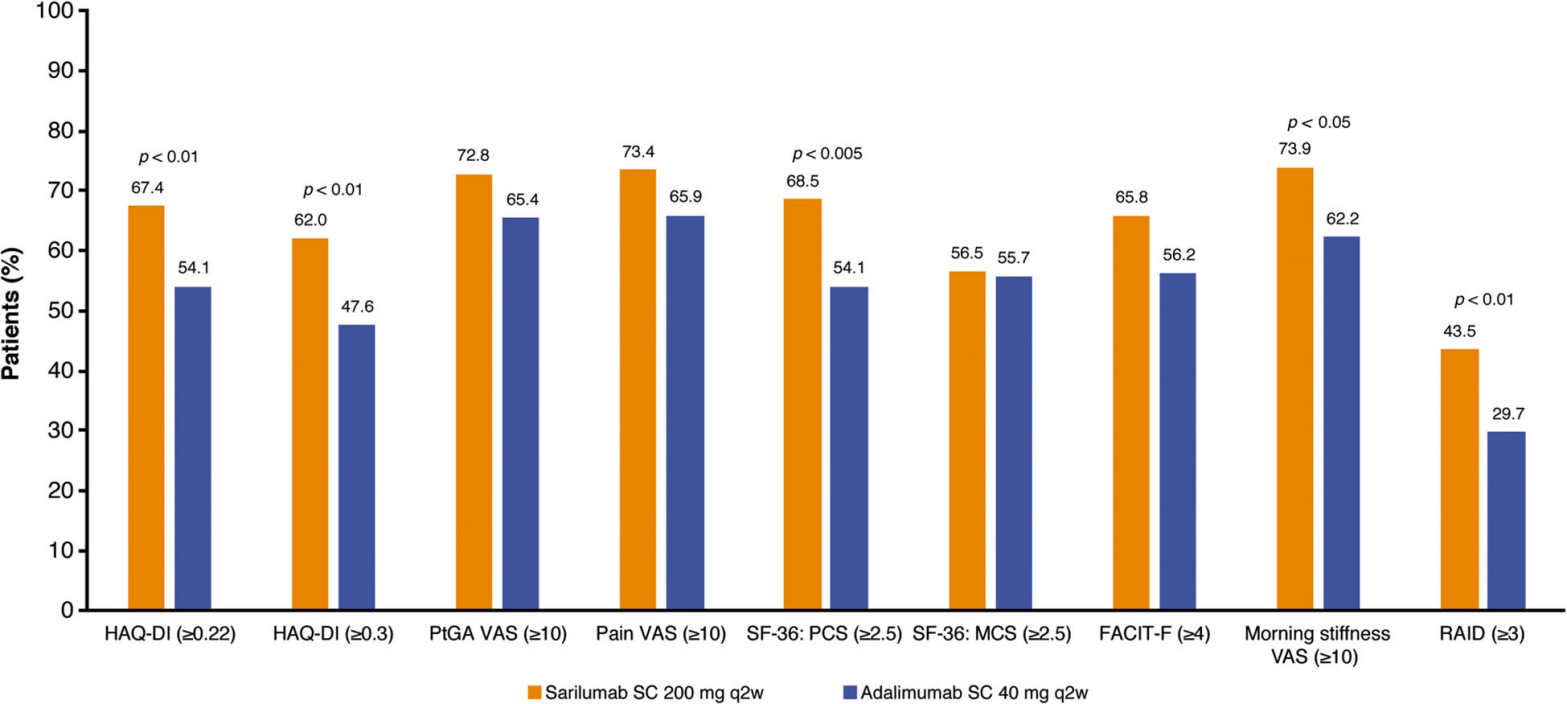
The percentage of patients reporting improvements greater than or equal to the minimal clinically important difference at week 24 with sarilumab 200 mg compared with adalimumab 40 mg every 2 weeks. All *p* values are nominal (between-group differences [sarilumab vs. adalimumab]). *FACIT-F* Functional Assessment of Chronic Illness Therapy–Fatigue, *HAQ-DI* Health Assessment Questionnaire Disability Index, *LSM* Least-squares mean, *MCS* Mental Component Summary, *PCS* Physical Component Summary, *PtGA* Patient global assessment of disease activity, *q2w* Every 2 weeks, *RAID* Rheumatoid Arthritis Impact of Disease, *SC* Subcutaneous, *SF-36* 36-item Short Form Health Survey, *VAS* Visual analogue scale

At week 24, improvements greater than or equal to the MCID were reported by a greater percentage of patients with sarilumab than adalimumab for HAQ-DI (67.4% vs. 54.1%; nominal *p* < 0.01 [≥ 0.22 units] and 62.0% vs. 47.6%; nominal *p* < 0.01 [≥ 0.3 units]), SF-36 PCS score (68.5% vs. 54.1%; nominal *p* < 0.005), RAID (43.5% vs. 29.7%; nominal *p* < 0.01), and morning stiffness (73.9% vs. 62.2%; nominal *p* < 0.05) (Fig. 2).

**Fig. 3:**
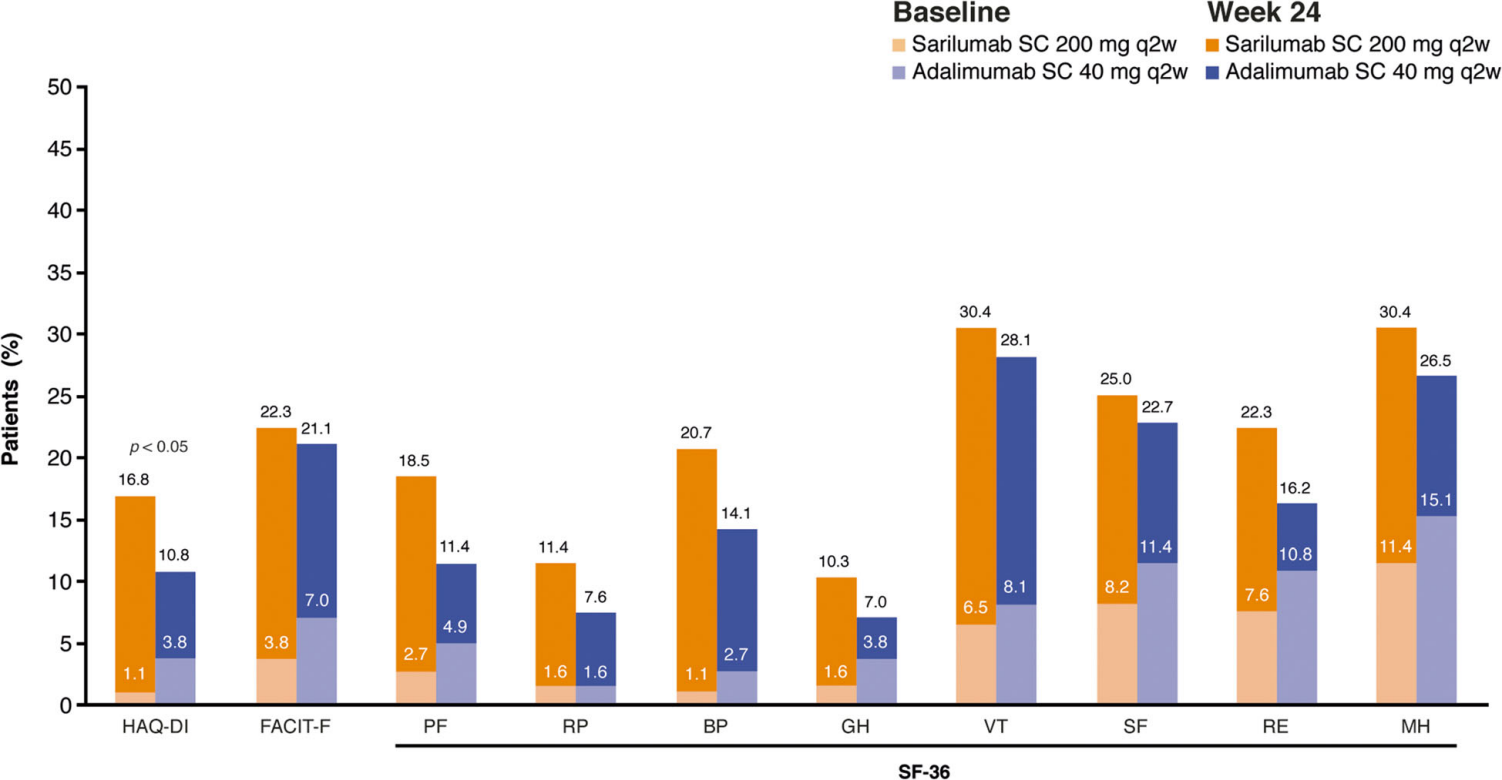
Percentage of patients reporting scores greater than or equal to normative values on Health Assessment Questionnaire Disability Index (HAQ-DI), Functional Assessment of Chronic Illness Therapy–Fatigue (FACIT-F), and 36-item Short Form Health Survey (SF-36) at baseline and week 24. ^†^LSM between-group differences (sarilumab vs. adalimumab). Nominal *p* value. *BP* Bodily pain, GH General health, *MCS* Mental Component Summary, *MH* Mental health, *PCS* Physical Component Summary, *PF* Physical functioning, *q2w* Every 2 weeks, *RE* Role emotional, *RP* Role physical, *SC* Subcutaneous, *SF* Social functioning, *VT* Vitality

As shown in Fig. 1, mean baseline scores for SF-36 domains were substantially below age- and sex-matched norms. The percentages of patients who reported normative values at baseline in HAQ-DI, FACIT-F, and individual SF-36 domains ranged from 1.1% for HAQ-DI and the SF-36 BP domain to 15.1% for SF-36 MH domain (Fig. 3). At week 24, the percentages of patients reporting scores greater than or equal to normative values in HAQ-DI, FACIT-F, and SF-36 PCS and MCS and all individual SF-36 domains increased with both sarilumab and adalimumab, with numerically greater increases reported with sarilumab than with adalimumab (Fig. 3).

## Discussion

These results from the MONARCH study show that sarilumab monotherapy is superior to adalimumab monotherapy according to a number of patient-reported endpoints, including physical function. Improvements were demonstrated by week 12, and between-group differences increased over time, despite the allowance of adalimumab or matched placebo (but not sarilumab) dose escalation in patients with poor responses. Results from these PRO analyses support the clinical efficacy data previously reported [14].

As in previous clinical trials of sarilumab and adalimumab [11, 13, 37], baseline PRO scores indicate a substantial impairment of general health status, physical function, and participation when compared with an age- and sex-matched U.S. general population. Considerable improvements in PROs were reported after treatment with both sarilumab and adalimumab. This is consistent with previous RCTs of sarilumab administered in combination with csDMARDs in MTX-IR and tumor necrosis factor inhibitor (TNFi)-IR RA populations [11, 13] and of adalimumab administered in combination with MTX in patients with early RA or as a monotherapy in patients with active RA who are intolerant of or inappropriate for continued MTX treatment [37, 38]. Notably, the proportion of patients reporting normative values in HAQ-DI, FACIT-F, and SF-36 PCS and MCS and all individual SF-36 domains was substantially increased with both therapies, with numerically greater increases reported with sarilumab than with adalimumab. These data indicate that attainment of normative values is now a reasonable goal for RA therapy.

Compared with adalimumab, sarilumab resulted in significantly greater improvements in PtGA, physical function, and indicators of health status (HAQ-DI, SF-36 PCS) and pain. Sarilumab monotherapy also resulted in greater improvements in RAID than adalimumab monotherapy, providing further evidence of the broad benefits of sarilumab monotherapy in reducing the impact of RA on patients’ lives. RAID is a relatively new disease-specific measure that provides a weighted profile of seven patient-valued domains: pain, functional disability, fatigue, sleep, physical/emotional well-being, and coping [26]. Improvements in RAID with sarilumab monotherapy are consistent with those previously reported with sarilumab plus csDMARDs in a TNFi-IR RA population [13].

The impact of fatigue is a key consideration for patients with active RA [39, 40]. Improvements in fatigue based on FACIT-F, the VT domain of SF-36, and the fatigue domain of RAID were reported in both the sarilumab and adalimumab groups. Improvements in the fatigue domain of RAID, FACIT-F, and SF-36 VT were numerically greater with sarilumab than with adalimumab; however, between-group differences in FACIT-F, which was part of the hierarchy, did not reach statistical significance. Morning stiffness has also been shown to have a significant impact on the lives and well-being of patients with RA and can result in frustration, distress, and absenteeism [41]. Both sarilumab and adalimumab resulted in improvements in morning stiffness [11, 13, 42, 43], with greater improvements with sarilumab than with adalimumab.

WPS-RA scores demonstrated that the interference of RA with work inside and outside the home and participation in family, social, and leisure activities were reduced with both sarilumab and adalimumab. Improvements in the global WPS-RA score were greater with sarilumab than with adalimumab. However, numerical between-group differences in the elements of the score that assess the impact of RA on work outside the home were small, although only 40% of the study population were employed, which may limit the ability to detect a difference.

The clinical efficacy data from the MONARCH trial are consistent with those of the ADACTA trial, which demonstrated superior clinical efficacy of tocilizumab monotherapy, another IL-6 receptor inhibitor, compared with adalimumab monotherapy, despite the allowance of adalimumab (or matched placebo) dose escalation [38]. However, in ADACTA, between-group differences in HAQ-DI and SF-36 PCS did not reach statistical significance, whereas improvements in SF-36 MCS were greater with tocilizumab than with adalimumab [38]. In MONARCH, sarilumab resulted in statistically significant and clinically meaningful improvements in HAQ-DI and SF-36 PCS compared with adalimumab, but between-group differences in SF-36 MCS were not significantly different.

PROs are an essential supplement to clinical data and provide a true measurable insight into treatment effects that are not completely assessed by clinical endpoints included in the ACR core set. Key strengths of this head-to-head trial are the wide range of PROs evaluated, including general and specific measures, which allow for a comprehensive evaluation of patient-reported benefits of two different bDMARD monotherapies, and the range of analyses undertaken to understand the level of benefit observed from a patient perspective. One limitation of the study was that several of the PROs were not included in the formal statistical testing hierarchy, which may limit the conclusions that can be made.

## Conclusions

In patients with RA who are unsuitable candidates for treatment with MTX owing to intolerance or inadequate response, treatment with sarilumab monotherapy resulted in greater patient-reported improvements in PtGA, pain, HAQ-DI, SF-36 PCS and PF, RP, BP and SF domain scores, morning stiffness, RAID, and WPS-RA compared with adalimumab monotherapy. Reducing the impact of RA on patients’ lives is an important treatment objective, and these data indicate that sarilumab monotherapy may result in better patient-reported benefits than monotherapy with a widely used bDMARD, adalimumab.

## Additional file

Additional file 1: **Table S1.** LSM changes in RAID individual domain scores from baseline to week 24 with sarilumab 200 mg or adalimumab 40 mg q2w. **Table S2.** LSM change from baseline to week 12 with sarilumab 200 mg or adalimumab 40 mg q2w. (DOCX 42 kb)

## Abbreviations

ACR: American College of Rheumatology
AE: Adverse event
bDMARD: Biologic disease-modifying antirheumatic drug
BP: Bodily pain
csDMARD: Conventional synthetic disease-modifying antirheumatic drug
DAS28-CRP: 28-Joint Disease Activity Score using C-reactive protein
DAS28-ESR: 28-Joint Disease Activity Score using erythrocyte sedimentation rate
DMARD: Disease-modifying antirheumatic drug
ESR: Erythrocyte sedimentation rate
FACIT-F: Functional Assessment of Chronic Illness Therapy–Fatigue
GH: General health
HAQ-DI: Health Assessment Questionnaire Disability Index
HRQOL: Health-related quality of life
IL-6: Interleukin-6
ITT: Intention to treat
LSM: Least-squares mean
MCID: Minimal clinically important difference
MCS: Mental Component Summary
MH: Mental health
MTX: Methotrexate
PCS: Physical Component Summary
PF: Physical functioning
PRO: Patient-reported outcome
PtGA: Patient global assessment of disease activity
q2w: Every 2 weeks
RA: Rheumatoid arthritis
RAID: Rheumatoid Arthritis Impact of Disease score
RE: Role emotional
RP: Role physical
SF: Social functioning
SF-36: 36-Item Short Form Health Survey
TNFi: Tumor necrosis factor inhibitor
VAS: Visual analogue scale
VT: Vitality
WPS-RA: Rheumatoid arthritis-specific Work Productivity Survey
