# Supplementary material for: Patient-reported outcomes from a randomized phase III trial of sarilumab monotherapy versus adalimumab monotherapy in patients with rheumatoid arthritis
Source: Arthritis Res Ther. 2018 Jun 19;20:129. doi: 10.1186/s13075-018-1614-z (PMC6009058; doi:10.1186/s13075-018-1614-z)
Supplement: Supplementary file 1 — Table S1. LSM changes in RAID individual domain scores from baseline to week 24 with sarilumab 200 mg or adalimumab 40 mg q2w. Table S2. LSM change from baseline to week 12 with sarilumab 200 mg or adalimumab 40 mg q2w. (DOCX 42 kb) [file 13075_2018_1614_MOESM1_ESM.docx]

Table S1 LSM changes in RAID individual domain scores from baseline to Week 24 with sarilumab 200 mg or adalimumab 40 mg q2w

| **RAID individual domain score** | **LSM (SE) change from baseline to Week 24** | | **LSM between-group difference (95% CI)** | ***p*-value^†^** |
| --- | --- | --- | --- | --- |
|  | **Sarilumab SC 200 mg q2w (n = 184)** | **Adalimumab SC 40 mg q2w (n = 185)** |  |  |
| Pain | –3.22 (0.18) | –2.34 (0.18) | –0.87 (–1.36, –0.39) | ≤0.01 |
| Functional disability | –3.12 (0.19) | –2.25 (0.19) | –0.87 (–1.38, –0.37) | ≤0.01 |
| Fatigue | –3.02 (0.20) | –2.33 (0.20) | –0.69 (–1.22, –0.15) | ≤0.05 |
| Sleep difficulties | –2.94 (0.21) | –2.23 (0.21) | –0.70 (–1.25, –0.14) | ≤0.05 |
| Physical well-being | –3.13 (0.19) | –2.39 (0.19) | –0.74 (–1.26, –0.22) | ≤0.01 |
| Emotional well-being | –2.65 (0.20) | –2.07 (0.20) | –0.58 (–1.12, –0.04) | ≤0.05 |
| Coping | –2.56 (0.19) | –1.92 (0.19) | –0.64 (–1.15, –0.12) | ≤0.05 |

*CI* confidence interval; *LSM* least squares mean; *q2w* every 2 weeks; RAID RA Impact of Disease*; SC* subcutaneous; *SE* standard error

^†^LSM between-group differences (sarilumab vs. adalimumab). All *p*-values are nominal.

Table S2 LSM change from baseline to Week 12 with sarilumab 200 mg or adalimumab 40 mg q2w

| **Patient-reported outcome** | **LSM (SE) change from baseline to Week 12** | | **LSM between-group difference (95% CI)** | ***p*-value^†^** |
| --- | --- | --- | --- | --- |
|  | **Sarilumab SC 200 mg q2w (n = 184)** | **Adalimumab SC 40 mg q2w (n = 185)** |  |  |
| HAQ-DI | –0.50 (0.04) | –0.35 (0.04) | –0.15 (–0.26, –0.04) | <0.050 |
| Pain VAS | –31.99 (1.84) | –26.47 (1.85) | –5.51 (–10.53, –0.49) | <0.050 |
| PtGA | –26.77 (1.69) | –22.44 (1.70) | –4.33 (–8.95, 0.29) | 0.066 |
| SF-36 component summaries |  |  |  |  |
| SF-36: PCS | 7.07 (0.52) | 5.32 (0.52) | 1.75 (0.36, 3.14) | <0.050 |
| SF-36: MCS | 6.12 (0.72) | 5.96 (0.72) | 0.16 (–1.78, 2.10) | 0.871 |
| SF-36 individual domains |  |  |  |  |
| Physical functioning | 18.66 (1.49) | 11.86 (1.49) | 6.80 (2.79, 10.80) | <0.001 |
| Role physical | 15.03 (1.47) | 14.28 (1.47) | 0.75 (–3.19, 4.68) | 0.709 |
| Bodily pain | 21.61 (1.45) | 17.70 (1.45) | 3.91 (0.02, 7.80) | <0.050 |
| General health | 10.95 (1.14) | 10.14 (1.14) | 0.82 (–2.24, 3.87) | 0.599 |
| Vitality | 14.63 (1.36) | 12.62 (1.35) | 2.02 (–1.62, 5.66) | 0.276 |
| Social functioning | 15.54 (1.64) | 14.58 (1.64) | 0.97 (–3.43, 5.36) | 0.665 |
| Role emotional | 12.81 (1.68) | 11.40 (1.68) | 1.41 (–3.09, 5.90) | 0.538 |
| Mental health | 12.61 (1.29) | 11.33 (1.28) | 1.29 (–2.17, 4.74) | 0.465 |
| FACIT-F | 8.66 (0.68) | 7.27 (0.68) | 1.39 (–0.42, 3.20) | 0.132 |
| Morning stiffness VAS | –32.15 (2.01) | –26.37 (2.00) | –5.79 (–11.17, –0.41) | <0.050 |
| RAID | –2.67 (0.16) | –2.18 (0.16) | –0.49 (–0.91, –0.07) | <0.050 |
| WPS-RA^‡^ | N/A | N/A | N/A | <0.050 |
| Work days missed^§^ | –0.51 (0.49) | –0.19 (0.56) | –0.32 (–1.66, 1.03) | 0.644 |
| Days with work productivity reduced by ≥50%^§^ | –3.07 (0.67) | –2.18 (0.77) | –0.89 (–2.74, 0.96) | 0.343 |
| Interference with work productivity^‖^ | –2.67 (0.30) | –1.92 (0.35) | –0.75 (–1.59, 0.09) | 0.078 |
| Housework days missed | –4.13 (0.49) | –3.34 (0.50) | –0.78 (–2.11, 0.55) | 0.247 |
| Days with household productivity reduced by ≥50% | –5.88 (0.50) | –4.56 (0.50) | –1.32 (–2.66, 0.03) | 0.055 |
| Interference with household productivity | –2.84 (0.21) | –2.52 (0.21) | –0.32 (–0.88, 0.24) | 0.257 |
| Days with family, social, or leisure activities missed | –3.29 (0.41) | –3.25 (0.41) | –0.04 (–1.15, 1.06) | 0.938 |
| Days with outside help hired | –2.81 (0.42) | –2.35 (0.42) | –0.46 (–1.59, 0.66) | 0.420 |
|  |  |  |  |  |

*ACR* American College of Rheumatology; *CI* confidence interval; *FACIT-F* Functional Assessment of Chronic Illness Therapy-Fatigue; *HAQ-DI* Health Assessment Questionnaire Disability Index; *LSM* least squares mean; *MCS* mental component summary; *PCS* physical component summary; *PtGA* Patient Global Assessment of Disease Activity; *q2w* every 2 weeks; *RAID* RA Impact of Disease; *SC* subcutaneous; *SE* standard error; *SF-36* 36-Item Short Form Survey; *VAS* visual analog scale; *WPS-RA* RA-specific Work Productivity Survey

^†^LSM between-group differences (sarilumab vs. adalimumab). All *p*-values are nominal.

^‡^Global test for the change from baseline in the 8 WPS-RA scores

^§^Number of patients included in the analysis for this element of the WPS-RA score: adalimumab group n = 60; sarilumab group n = 70

^‖^Number of patients included in this element of the WPS-RA score: adalimumab group n = 61; sarilumab group n = 68
